# Resistance to Pembrolizumab and Axitinib in Renal Cell Carcinoma: Clinical and Genomic Evaluation

**DOI:** 10.15586/jkcvhl.2020.135

**Published:** 2020-06-02

**Authors:** Panagiotis J. Vlachostergios

**Affiliations:** 1 Department of Medicine, Division of Hematology and Medical Oncology, Weill Cornell Medicine, New York, NY, USA; 2 Department of Medicine, Division of Hematology and Medical Oncology, New York Presbyterian Brooklyn Methodist Hospital, Brooklyn, NY, USA

**Keywords:** angiogenesis, molecular biomarker, mutation, immunotherapy, renal cell carcinoma

## Abstract

Clear cell renal cell carcinoma (ccRCC) represents the most common subtype of renal cell carcinoma (RCC). In spite of recent advances in the treatment armamentarium and outcomes with the combined use of immune checkpoint and angiogenesis inhibitors, prediction of responses and selection of patients remain a challenge. This is a case of ccRCC with recurrence to the liver 1 year following right radical nephrectomy, who rapidly progressed on frontline therapy with axitinib/pembrolizumab. The clinical course and targeted tumor sequencing findings are discussed. In addition to established clinical prognostication in RCC, several surrogate markers of efficacy or/and resistance have been proposed for immunotherapy or/and anti-angiogenic therapy. Since the majority of patients will still progress after these combinations, it is becoming increasingly important to develop robust predictive biomarkers to guide patient selection and sequencing of targeted therapies.

## Introduction

Recent advances in the frontline therapy of advanced renal cell carcinoma (RCC) have led to novel combinations of agents targeting programmed death-1/programmed death-1 ligand (PD-1/PD-L1) and the angiogenesis/vascular endothelial growth factor receptor (VEGFR) pathway. The anti PD-1 monoclonal antibody (Mab) pembrolizumab (anti-PD-1) and the anti-PD-L1 avelumab were both FDA approved, each in combination with the VEGFR tyrosine kinase inhibitor (TKI) axitinib for previously untreated patients with advanced RCC, after improving the objective response rate (ORR), progression-free survival (PFS), or/and overall survival (OS), compared to the prior standard, sunitinib (1–3). This benefit was observed across all International Metastatic Renal Cell Carcinoma Database Consortium risk groups (favorable, intermediate, and poor risk) (1–4). In addition, the combination of the cytotoxic T-lymphocyte-associated protein 4 (CTLA-4) inhibitor ipilimumab and the PD-1 inhibitor nivolumab was approved for intermediate- and poor-risk, patients with advanced RCC (5). Nevertheless, metastatic RCC remains a lethal disease overall, with a 5-year survival of approximately 10–20% (6), and a proportion of patients will still progress in spite of optimal therapy. At present, the majority of clinicians are not using any predictive biomarkers for treatment decision-making (7). A small proportion (10.9–12.4%) of advanced RCC patients treated with frontline PD-1/VEGFR-targeted combination therapy have demonstrated progressive disease as best response in the two major phase III studies of axitinib/pembrolizumab and axitinib/avelumab, respectively (1–3). The underlying biology of this intrinsic resistance is poorly understood. Herein, the clinical and genomic evaluation of a case of advanced refractory RCC with lack of response to first-line axitinib/pembrolizumab is presented. The aim of molecular analysis of a liver metastasis from this patient was to assess for somatic alterations that could potentially be indicators of primary resistance to the combination or/and sensitivity to other agents.

## Case Report

**Figure 1:**
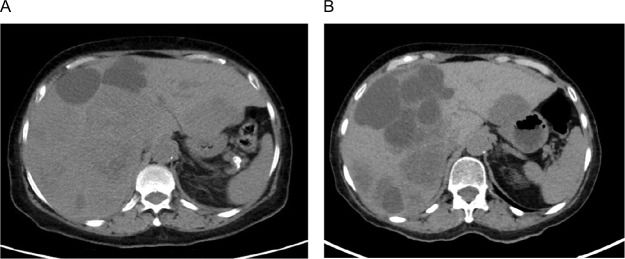
(A) Computed tomography (CT) abdomen and pelvis (pre-treatment) showing hepatomegaly and several hypodense lesions throughout the liver parenchyma consistent with liver metastases. (B) CT abdomen and pelvis (post-treatment) showing enlargement of existing lesions and new hepatic metastases.

A 70-year-old woman presented with worsening right upper quadrant pain of 2 weeks duration, associated with anorexia and 10 lb weight loss. She had a history of right nephrectomy 13 months earlier for a grade 3, stage III pT3aNxM0 clear cell renal cell carcinoma (ccRCC), with extension to the renal vein and uninvolved margins; however, she did not follow up after surgery. No family history of cancer was reported. A computed tomography (CT) scan of her abdomen and pelvis disclosed hepatomegaly and several hypodense lesions throughout the liver parenchyma consistent with liver metastases (Figure 1A). Labs were remarkable for a hemoglobin level of 11.2 g/dL and an elevated serum lactate dehydrogenase level of 612 U/L.

The patient was started on pembrolizumab 200 mg intravenously every 3 weeks and axitinib 5 mg orally twice daily as first-line therapy for her recurrent/metastatic disease after completing full restaging, which was otherwise negative. She required dose reduction of her axitinib to 3 mg twice daily due to fatigue, and experienced elevated thyroid stimulating hormone (TSH) of 16.5 mcIU/mL (normal free T4), which improved on levothyroxine. Unfortunately, after completing 12 weeks on combination axitinib/pembrolizumab, repeat CT scans for assessment of response disclosed progression of liver metastases (Figure 1B).

A left hepatic lobe lesion was biopsied under CT guidance. Four core specimens were obtained. Deoxyribonucleic acid (DNA) and ribonucleic acid (RNA) were extracted from macrodissected, paraffin-embedded tumor of the patient using the Maxwell 16 instrument (Promega, Madison, WI) and RecoverAll Total Nucleid Acid Isolation Kit (Life Technologies, Walltham, MA), respectively. The extracted DNA and synthesized complementary DNA (cDNA) from the extracted RNA were amplified by the Oncomine Comprehensive Panel (OCP) and subjected to Next Generation Sequencing (NGS) using the Ion Torrent S5^TM^ (Life Technologies), as previously described (8). OCP was developed, and its performance characteristics were determined by the Clinical Genomics Laboratory, Englander Institute for Precision Medicine, Department of Pathology and Laboratory Medicine at Weill Cornell Medicine, New York-Presbyterian Hospital. OCP is approved by the New York State Department of Health (NYS-DOH). Targeted tumor sequencing of patient’s CT-guided liver biopsy showed loss of function (pathogenic) mutations in von Hippel-Lindau (VHL) and BRCA1-associated protein (BAP1) genes, as well as copy-number loss of cyclin-dependent kinase inhibitor 2A (CDKN2A) (Table 1). Tumor purity (neoplastic content) of the tissue analyzed was 80%.

**Table 1:** Genomic alterations (pathogenic) of liver metastasis from clear cell renal cell carcinoma.

Gene	Alteration	Type	VAF
VHL	c.162_163insCG, p.Glu55Argfs*13	Mutation – frameshift insertion	32.8%
BAP1	c.509T>G, p.Phe170Cys	Mutation – missense	42.6%
CDKN2A	loss	Copy number alteration – loss	N/A

VAF: variant allele frequency, N/A: not applicable, VHL, von Hippel-Lindau, BAP1, BRCA1-associated protein, CDKN2A, cyclin-dependent kinase inhibitor 2A.

## Discussion

Resistance to systemic therapies in advanced RCC, either intrinsic due to presence of resistant clones or acquired after initial tumor regression can directly impact the clinical course and additional treatment approach of these patients in contemporary practice.

While the underlying mechanisms are a field of ongoing investigation, numerous studies have identified molecular alterations in primary and metastatic RCC tumors that may be contributing to the development of resistance (9).

The VHL gene is the most frequently mutated gene in the majority (80–90%) of sporadic RCCs (9–11). Mechanistically, the loss of VHL protein function leads to the accumulation of hypoxia-inducible factor (HIF) that promotes angiogenesis and tumor growth (9, 11). It has been found that the effect of VHL mutation on responses to VEGFR TKIs in patients with metastatic ccRCC is minimal, if any (12). Furthermore, although VHL is an inducer of PD-L1 through upregulation of HIF-2α (13), PD-L1 immunohistochemical expression status had no effect on ORR and PFS in neither of the two large phase 3 trials testing the PD-1/PD-L1 plus VEGFR pathway inhibitors combination (1–3). Other studies have demonstrated that overexpression and activation of the receptor tyrosine kinases MET and AXL due to VHL inactivation is implicated in resistance to VEGFR-targeted therapies (14). Specifically, combination of axitinib with the c-met inhibitor crizotinib in RCC patient-derived xenograft models resulted in decreased tumor microvessel density, growth inhibition, and improved survival (15).

The BAP1 gene is mutated in different cancers, including ccRCC in up to 11% of cases (10). The resultant loss of function of this tumor suppressor was associated with more aggressive morphologic features (16) and decreased OS both in The Cancer Genome Atlas (TCGA) RCC cohort and within the ccRCC group (10). Besides its prognostic relevance, BAP1 loss in non-RCC tumors was correlated with upregulation of suppressive immune gene responses, for example, HLA-DR, CD38, and CD74 (uveal melanoma) (17), and the promotion of an inflammatory tumor microenvironment (peritoneal mesothelioma) (18). Interestingly, an integrated biomarker analysis of 412 RCC patients who were treated on the phase 3 COMPARZ trial comparing pazopanib versus sunitinib (19) demonstrated that those with tumors harboring mutated BAP1 had inferior OS compared to wild-type ones (20). Angiogenesis gene expression scores from a 43-gene signature were lower in patients with BAP1-mutated tumors, suggesting that BAP1 loss correlates with lower angiogenic signaling and worse outcomes after treatment with TKIs (20). Overall, both BAP1 and VHL deleterious/loss of function mutations were identified in hyperprogressive tumors post anti-PD-1 treatment, suggesting a potential involvement in the development of resistance (21).

CDKN2A is a ubiquitously expressed gene that encodes two other key genes, the INK4 family member p16 (p16INK4a) and p14ARF, both of which act as tumor suppressors regulating the cell cycle. CDKN2A gene aberrations, including mutations, hypermethylation, or deletions, occur in approximately 16% of ccRCCs (10), with loss of the chromosome 9p region encoding CDKN2A being the most frequent event (12%) in these cases (10). CDKN2A loss is associated with a higher tumor stage at diagnosis (22) and predicts shorter OS across all TCGA RCC subtypes (10). Patients with tumors that harbor chromosomal CDKN2A loss are frequently characterized by resistance to immunotherapy, which has been mechanistically explained, at least partially, by a concurrent deletion of the interferon-gamma (IFNγ) signaling pathway gene JAK2 (23). In addition, copy-number variations of CDKN2A or/and other genes in the CDK4 pathway (CDK4, CCND1) are associated with innate resistance to anti-PD-1 therapy in patients with advanced melanoma, which can be reversed with addition of CDK4/6 inhibitors to anti-PD-1 antibodies (24). Interestingly, CDK4/6 inhibitors have shown activity in RCC *in vitro*, and the combination of abemaciclib with sunitinib resulted in a dramatic reduction in tumor sizes in a RCC mouse model (25). A phase 1 trial of these two agents is currently ongoing in progressing patients with metastatic ccRCC (NCT03905889).

Collectively, treatment of RCC patients with disease progression after axitinib/pembrolizumab or axitinib/avelumab is complex, and the current expert consensus agreement is on the use of another VEGFR pathway inhibitor, cabozantinib, which also has activity against MET, AXL, and RET kinases (7). A potential predictive value of T-effector gene expression and angiogenesis signatures has been supported from exploratory biomarker analyses of PD-1/PD-L1 plus VEGFR TKI combination studies (26–28); however, this is largely hypothesis-generating and not ready for clinical use. It also remains ill-defined as to how potential genomic biomarkers could be combined with the established clinical prognostication tools (the IMDC, the Cleveland Clinic Foundation CCF model, the International Kidney Cancer Working Group IKCWG model, the French model, and the Memorial Sloan-Kettering Cancer Center model) (4) to improve our prediction of effective therapies. In phase 2 and 3 studies of the anti-PD-L1 Mab atezolizumab combined with the anti-VEGF-A Mab bevacizumab, expression of angiogenesis genes was enriched in patients with favorable risk, compared to those with intermediate and poor risk according to the MSKCC risk stratification model (28).

## Conclusion

This case of advanced refractory RCC with clinical and genomic evaluation highlights an unmet need for better characterizing the underlying biology of treatment-resistant RCC in order to improve our ability to conduct biology- and biomarker-driven trials to help guide selection of the right drug, for the right target, in the right patient, at the right time.
